# Inclusion of mobile phone numbers into an ongoing population health survey in New South Wales, Australia: design, methods, call outcomes, costs and sample representativeness

**DOI:** 10.1186/1471-2288-12-177

**Published:** 2012-11-22

**Authors:** Margo L Barr, Jason J van Ritten, David G Steel, Sarah V Thackway

**Affiliations:** 1 Centre for Epidemiology and Evidence, NSW Ministry of Health, 73 Miller Street, North Sydney, Australia; 2 Centre for Statistical and Survey Methodology, University of Wollongong, Wollongong, Australia

**Keywords:** Sample survey, Mobile phone, Sampling frame

## Abstract

**Background:**

In Australia telephone surveys have been the method of choice for ongoing jurisdictional population health surveys. Although it was estimated in 2011 that nearly 20% of the Australian population were mobile-only phone users, the inclusion of mobile phone numbers into these existing landline population health surveys has not occurred. This paper describes the methods used for the inclusion of mobile phone numbers into an existing ongoing landline random digit dialling (RDD) health survey in an Australian state, the New South Wales Population Health Survey (NSWPHS). This paper also compares the call outcomes, costs and the representativeness of the resultant sample to that of the previous landline sample.

**Methods:**

After examining several mobile phone pilot studies conducted in Australia and possible sample designs (screening dual-frame and overlapping dual-frame), mobile phone numbers were included into the NSWPHS using an overlapping dual-frame design. Data collection was consistent, where possible, with the previous years’ landline RDD phone surveys and between frames. Survey operational data for the frames were compared and combined. Demographic information from the interview data for mobile-only phone users, both, and total were compared to the landline frame using χ^2^ tests. Demographic information for each frame, landline and the mobile-only (equivalent to a screening dual frame design), and the frames combined (with appropriate overlap adjustment) were compared to the NSW demographic profile from the 2011 census using χ^2^ tests.

**Results:**

In the first quarter of 2012, 3395 interviews were completed with 2171 respondents (63.9%) from the landline frame (17.6% landline only) and 1224 (36.1%) from the mobile frame (25.8% mobile only). Overall combined response, contact and cooperation rates were 33.1%, 65.1% and 72.2% respectively. As expected from previous research, the demographic profile of the mobile-only phone respondents differed most (more that were young, males, Aboriginal and Torres Strait Islanders, overseas born and single) compared to the landline frame responders. The profile of respondents from the two frames combined, with overlap adjustment, was most similar to the latest New South Wales (NSW) population profile.

**Conclusions:**

The inclusion of the mobile phone numbers, through an overlapping dual-frame design, did not impact negatively on response rates or data collection, and although costing more the design was still cost-effective because of the additional interviews that were conducted with young people, Aboriginal and Torres Strait Islanders and people who were born overseas resulting in a more representative overall sample.

## Background

Because of increasing numbers of mobile-only phone users worldwide, currently estimated to be 30.2% in the USA [1], 13% in Canada [2], 14% - 19% across the UK countries [3] and 19% in Australia [4], it has become increasingly difficult to produce unbiased estimates from random digit dialling (RDD) surveys that only target landline phones [5-7]. Consequently there is now substantial international literature on conducting RDD surveys with mobile phone augmentation [8-12] and the American Association for Public Opinion Researchers (AAPOR) Cell Phone Task Force recommended in their latest report (2010) [12]: “Random digit dialling (*RDD) surveys without cell phone augmentation should in their methods report how they have produced unbiased estimates without the cell phone only segment”.*

In Australia landline telephone surveys have been the method of choice for ongoing population health surveys [13-18]. Although the rate of mobile-only phone users was estimated to be nearly 20% in 2011 [4] the inclusion of mobile-only phone users into these existing landline population health surveys has not occurred. Studies describing the demographic, socio-economic and health profile of mobile-only phone users have been conducted and have shown that mobile-only phone respondents were different to those who had access to a landline phone using face-to-face survey data [19,20] and internet panel data [21].

Two designs for the inclusion of mobile-only phone users into landline RDD surveys have been discussed in the literature: screening dual-frame design and overlapping dual-frame design [6]. The screening dual-frame design attempts to remove any overlap units usually by screening for telephone ownership prior to conducting the survey and then only interviewing mobile-only phone users from the mobile frame. The overlapping dual-frame design accounts for the overlap in the weighting by using an average estimator and a compositing factor. The overlapping dual-frame design, although requiring a more complex weighting strategy, has been growing in favour because it has been shown that persons selected through mobile frames (even if they have both mobile and landline phones) differ to persons selected through landline frames [7].

Two pilots using a dual-frame design had also been conducted in Australia by Pennay in 2010 (700 respondents) and Lui et al. in 2011 (335 females respondents aged 18 to 39 years) [22,23]. Pennay [22] provided particularly useful statistics for planning this study including: the expected numbers of telephone numbers required to get an interview in each of the frames (landline 12 numbers and mobile 25 numbers) and the expected percentage of interviews with persons from landline-only phone households in the landline phone frame (14.5%), and percentage of interviews with mobile-only phone users from the mobile phone frame (27.6%).

This paper describes the methods used for the inclusion of mobile phone numbers into the New South Wales Population Health Survey (NSWPHS), an existing ongoing landline RDD health survey in an Australian state [13]. This paper also compares the call outcomes, costs and the representativeness of the resultant sample to that of the previous landline sample.

## Methods

### Survey methodology

Since 2002 the health and wellbeing of the New South Wales (NSW) population (7.3 million) has been monitored using the NSWPHS. A representative sample of approximately 15,000 persons are interviewed each year, with equal numbers from each of the strata (health administrative areas) using landline RDD computer assisted telephone interviewing (CATI). The questionnaire includes questions on: health behaviours, health status, social determinants, demographics and phone ownership. The survey has approval from the NSW Population and Health Services Research Ethics Committee. The questionnaires and the data collection methods are available on the survey website [13].

In order to include mobile only phone users into this existing landline RDD health survey an overlapping dual-frame design was chosen. This allowed us to examine the representation of the resultant sample for both an overlapping dual-frame design and, by excluding persons with both mobile phones and landline phones from the mobile frame, a screening dual-frame design.

Details about the procedures for sample generation, sample design, eligibility, sample size, questionnaire, data collection, calling protocol, participant selection and probability of selection weighting for the previous years’ landline RDD surveys [24-27] as well as for each of the phone frames are shown in Table 1. As shown in Table 1 the procedures were, where possible, consistent with the previous years’ landline RDD surveys and between frames.

**Table 1:** Comparison of survey methods, 2011 NSW Population Health Survey and 2012 NSW Population Health Survey

**Procedures and Protocols**	**2011 NSW Population Health Survey (*Landline phone numbers)***	**2012 NSW Population Health Survey**	
		***Landline phone numbers***	***Mobile phone numbers***
**Sample generation**	Landline RDD sample frame for each of the administrative strata were generated using “best fit” postcodes for the geography (exchange district and charge zone) associated with the Australian Communications and Media Authority (ACMA) phone number ranges for NSW [25]. The sample was then randomly ordered within each strata and each number was tested using proprietary software [26] to identify valid and invalid numbers. The resulting valid numbers were used for the study.	***Same as for previous landline survey***	The RDD mobile sample frame was developed using all known Australian mobile prefixes and then using proprietary software [27] each number was tested to identify valid and invalid numbers. A random sample of valid mobile numbers was then provided for the study.
**Sample design**	Stratified two-stage cluster sample design, with: strata defined by health administration areas; simple random sampling of clusters (household telephone numbers) within each stratum; and simple random sampling of population elements (household residents) within each cluster.	***Same as for previous landline survey***	Two-stage cluster sample design with simple random sampling of the mobile telephone numbers (adult population element) and simple random sampling of children in household (child population elements).
**Questionnaire**	The questionnaire included questions on: health behaviours, health status, social determinants, demographics (including number of adults and children in the household) and landline phone ownership ("How many residential telephone numbers do you have? Do not include mobile phone numbers or dedicated FAX numbers or modems."). The actual questions in the questionnaire are available on the survey website.	***Same as for previous landline survey except for*** the addition of two questions on mobile phone ownership ("How many mobile phone numbers do you personally have?" and "Is/are your residential telephone number/s listed in the White pages?")	***Same as for previous landline survey*** the addition of two questions on mobile phone ownership ("How many mobile phone numbers do you personally have?" and "Is/are your residential telephone number/s listed in the White pages?")
**Sample**	3000 persons per quarter with equal numbers in each of the strata	2000 persons per quarter	1000 persons per quarter
**Ineligible**	Business landline numbers, non-NSW residential numbers	***Same as for previous landline survey***	Business mobile numbers, non-NSW residential mobile numbers or mobile numbers owned by a child under the age of 16 years.
**Data collection**	Data collection was undertaken using SAWTOOTH WinCati version 4.2 and trained interviewers from the in-house NSW Ministry of Health’s CATI facility.	***Same as for previous landline survey***	***Same as for previous landline survey***
**Calling protocol**	The interviewers rang the randomly ordered landline numbers consecutively to try and contact households and convince the household and the respondent to participate in the survey. Up to 12 attempts were made to establish contact and if possible secure an interview with the selected respondent within a household.	***Same as for previous landline survey***	The interviewers rang the randomly ordered mobile phone numbers consecutively to try and contact the owner of the phone. Because mobile numbers could be located anywhere in Australia initial calls were timed to accommodate different time zones across Australia. Up to 12 attempts were made to establish contact and if possible secure an interview with the mobile phone holder.
**Participant selection**	One person from the household was randomly selected for inclusion in the survey. If the selected respondent was a child under the age of 16 years, a parent or carer completed the interview on their behalf.	***Same as for previous landline survey***	The mobile phone holder was selected. If the owner of the mobile phone was a parent of a child under 16 years of age they were asked at the end of the interview if they or the main carers would agree to being contacted at a later date to undertake an interview about one of their children chosen at random.
**Weighting (probability of selection)**	Adjust for differences in the probabilities of selection among subjects (using household size and number of landline phones in household).	***Same as for previous landline survey except for*** the inclusion of ratio of landline sample to landline phone populations for each strata.	Adjust for differences in the probabilities of selection among subjects (using number of mobile phones owned by respondent and ratio of mobile phone sample to mobile phone population and number of children in the household).

### Call outcomes and costing

Operational data for the survey were downloaded. The data included telephone number, number of attempts, details of each attempt (including duration) and final disposition. Although the final disposition codes used for the survey are site specific they can be easily mapped to the AAPOR definitions [28]. These final dispositions were then entered into the AAPOR outcome rate calculator [29] and all AAPOR levels of response, cooperation, refusal and contact rates were calculated from the groupings of the final dispositions for each frame. Overall rates were then calculated as described in the Non-response in RDD Cell phone surveys chapter of the AAPOR Cell Phone Task Force Report [12] using the latest ACMA figures for Australia (5% landline-only phone users, 19% mobile-only phone users, and 76% both mobile phone and landline phone users) [4].

The productivity (phone numbers to get a contact, an eligible contact, and an interview) of the sample for each frame was examined. Call costs (including connection fee, if applicable) and interviewer costs (hourly rate multiplied by the calling time) for each sample frame were also calculated and presented as a cost per completed interview.

### Demographic parameter comparisons

Interview data for the survey were downloaded. The data included a unique identifier, sample frame, strata, and responses to the health behaviours, health status and demographic questions. Demographic information from the mobile frame sample was compared to the landline frame sample using *χ*^2^ tests. Demographic information from the mobile frame sample, landline frame sample, combined landline sample with the mobile-only sample (equivalent to a screening dual frame design) and the combined landline sample and mobile sample with appropriate overlap adjustment was compared to the NSW demographic profile from the 2011 census using *χ*^2^ tests.

## Results

In the first quarter of 2012, 3395 interviews were completed with 2171 (63.9%) being from the landline frame of which 382 (17.6%) were landlines-only and 1224 (36.1%) being from the mobile frame of which 316 (25.8%) were mobile-only.

As shown in Table 2, completed interviews from the mobile frame, compared to the landline frame, were slightly shorter (15.6 minutes v 17.2 minutes), cost 2.3 times more for each completed interview ($74.42 v $31.13) and required more telephone numbers to obtain a contact (2.1 v 1.9), eligible contact (10.5 v 7.0) and an interview (14.4 v 9.8).

**Table 2:** Call outcome information and rates for by sample frame and overall (combined)

	**Landline frame**	**Mobile frame**	**Overall**
T=Total phone numbers used	21350	17534	38884
I=Complete Interviews (1.1)	2171	1224	3395
*Adults*	*1865*	*1085*	*2950*
*Children*	*306*	*139*	*445*
P=Partial	0	0	0
R=Refusal and break off (2.1)	868	457	1325
NC=Non Contact (2.2)	660	238	898
O=Other (2.0, 2.3)	1163	767	1930
e: estimated proportion of cases of unknown eligibility that are eligible.	*0.29*	*0.22*	*0.25*
UH=Unknown Household (3.1)	4553	5450	10003
UO=Unknown other (3.2-3.9)	0	0	0
NE=Not eligible	11935	9462	21397
*Fax data line (NE*_*F*_*)*	*1352*	*33*	*1385*
*Non-working number or unusual tone (NE*_*NW*_*)*	*2390*	*2637*	*5027*
*Business, government office, or other organizations(NE*_*B*_*)*	*8100*	*826*	*8926*
*Not in NSW or mobile owned by child (mobile frame)(NE*_*I*_*)*	*93*	*5966*	*6059*
***Survey length, collection costs and productivity***			
Average survey length (mins)	17.2	15.6	
Average call costs (per completed interview)	$7.45	$38.90	
Average interviewer time costs (per completed interview)	$23.68	$35.53	
Total average costs (call costs plus interviewer time costs)	$31.13	$74.42	
Telephone numbers used to get a contact: T/(I+R+NE_I_+NE_B_)	1.9	2.1	
Telephone numbers used to get an eligible contact: T/(I+R)	7.0	10.5	
Telephone numbers used to get a completed interview: T/I	9.8	14.4	
***Response Rates***			
Response Rate 1: I/(I+P) + (R+NC+O) + (UH+UO)	23.1%	15.0%	18.6%
Response Rate 2: (I+P)/(I+P) + (R+NC+O) + (UH+UO)	23.1%	15.0%	18.6%
Response Rate 3: I/((I+P) + (R+NC+O) + e(UH+UO) )	35.1%	31.5%	33.1%
Response Rate 4: (I+P)/((I+P) + (R+NC+O) + e(UH+UO) )	35.1%	31.5%	33.1%
***Cooperation Rates***			
Cooperation Rate 1: I/(I+P)+R+O)	51.7%	50.0%	50.7%
Cooperation Rate 2: (I+P)/((I+P)+R+O))	51.7%	50.0%	50.7%
Cooperation Rate 3: I/((I+P)+R))	71.4%	72.8%	72.2%
Cooperation Rate 4: (I+P)/((I+P)+R))	71.4%	72.8%	72.2%
***Refusal Rates***			
Refusal Rate 1: R/((I+P)+(R+NC+O) + UH + UO))	9.2%	5.6%	7.2%
Refusal Rate 2: R/((I+P)+(R+NC+O) + e(UH + UO))	14.0%	11.7%	12.8%
Refusal Rate 3: R/((I+P)+(R+NC+O))	17.9%	17.0%	17.4%
***Contact Rates***			
Contact Rate 1: (I+P)+R+O / (I+P)+R+O+NC+ (UH + UO)	44.6%	30.1%	36.5%
Contact Rate 2: (I+P)+R+O / (I+P)+R+O+NC + e(UH+UO)	68.0%	62.9%	65.1%
Contact Rate 3: (I+P)+R+O / (I+P)+R+O+NC	86.4%	91.1%	89.1%

Notes to Table 2: AAPOR Categories [28] are as follows: Interview (*I*) = Complete interviews (1.1); Refusal (*R*) = Respondent refusal (2.112), Household refusal and break off (2.1); Non-contact (*NC*) = Respondent never available(2.2), away for duration of survey (2.21); Other (*O*) = Respondent physically or mentally unable to complete interview (2.32), Non-translated language(2.333), Other non-refusal : hang up said nothing/ terminated by interviewer/technical problems (2.3); Unknown Household (*UH*) = Engaged busy (3.12), No answer (3.13), always answering machine (3.14); Not eligible (*NE*) = Fax data line (4.2), Non-working number (4.3), unusual tone (4.31), Business, government office, other organizations (4.51), Non-eligible respondent: not in NSW/mobile owned/answered by child (4.7);

Calculation of each rate for Overall = (R_A_* (N_a_+λN_ab_^A^))+ (R_B_ * (N_b_+(1-λ)N_ab_^B^)) where R frame rate; N population proportion; λ=overlap adjustment (set 0.5); _A_ landline sample frame; _B_ denotes mobile sample frame; _a_ landline-only phone users; _b_ mobile-only phone users; _ab_ denotes both mobile phone and landline users.

### Outcome rates

Levels of response, contact, cooperation and refusal rates, calculated as per AAPOR definitions, as shown in Table 2 were similar between frames. Overall combined (with adjustment for the overlap) response, contact, co-operation and refusal rates were 33.1%, 65.1%, 72.2% and 17.4% respectively.

### Sample characteristics

Table 3 shows respondent demographic profiles for the mobile frame (mobile-only, both and total), compared to the landline frame (landline-only, both and total). As shown in Table 3 the demographic profile of the landline frame responders was significantly different to respondents: from the mobile frame who were mobile-only for age group (p<0.001), sex (p=0.049), Aboriginality (p=0.049), country of birth (p<0.001), and marital status (p<0.001); from the mobile frame who had both mobile and landline phones for age group (p<0.001) marital status (p=0.003) and income (p=0.001); from the mobile frame for age group (p<0.001), country of birth (p<0.001), marital status (p<0.001) and income (p=0.01).

**Table 3:** Comparison of the demographic profile of the mobile frame and the landline frame respondents

**Demographic group**		**Mobile frame**						**Landline frame**		
		**Mobile only (%)**	**p-value**	**Both (%)**	**p-value**	**Total (%)**	**p-value**	**Land-line only (%)**	**Both (%)**	**Total (%)**
Age groups	0-15	8.5	**<0.001**	12.3	**<0.001**	11.4	**<0.001**	6.0	15.8	**14.1**
	16-24	17.1		10.8		12.4		0.5	4.9	**4.1**
	25-34	41.8		16.6		23.1		1.6	6.4	**5.6**
	35-44	12.3		16.0		15.0		5.2	8.0	**7.6**
	45-54	10.1		19.3		16.9		7.3	14.3	**13.0**
	55-64	7.3		14.9		12.9		16.8	22.6	**21.6**
	65-74	2.5		7.9		6.5		23.3	17.3	**18.4**
	75-high	0.3		2.2		1.7		39.3	10.6	**15.6**
Sex	Male	48.4	**0.049**	48.3	0.052	48.4	0.052	42.9	38.0	**38.9**
	Female	51.6		51.7		51.6		57.1	62.0	**61.1**
Aboriginality	Aboriginal	5.1	**0.049**	1.8	0.76	2.6	0.78	2.4	2.2	**2.2**
	Non-Aboriginal	94.9		98.2		97.4		97.6	97.8	**97.8**
Country of birth	Australia	60.8	**<0.001**	79.4	1.00	64.9	**<0.001**	76.6	80.1	**79.4**
	Overseas	39.2		20.6		35.1		23.4	19.9	**20.6**
Marital status	Married	31.3	**<0.001**	61.8	**0.003**	54.0	**<0.001**	45.3	56.0	**54.1**
	Widowed	1.9		3.5		3.1		28.7	10.5	**13.7**
	Separated	3.5		3.2		3.3		3.4	4.1	**4.0**
	Divorced	7.4		7.0		7.1		10.8	12.6	**12.3**
	Never married	55.8		24.5		32.5		11.8	16.8	**15.9**
Income	< $20,000	19.0	0.32	9.9	**0.001**	12.0	**0.01**	46.8	19.7	**24.0**
	$20,001-$40,000	14.7		15.7		15.4		24.5	18.9	**19.8**
	$40,001-$60,000	16.8		14.3		14.9		9.3	16.2	**15.1**
	$60,001-$80,000	14.2		13.9		14.0		4.1	11.5	**10.4**
	$80,000 plus	35.3		46.3		43.7		15.2	33.7	**30.8**

*Notes:* Chi-squared testing, setting the significance level of p<0.05, was used for the comparisons between the mobile phone frame (mobile-only, both and total) sample demographic categories and the total landline frame sample.

Table 4 shows respondent demographic profiles for the landline frame, mobile frame, the landline frame with the mobile-only respondents from the mobile frame, the combined frames (using λ=0.5 as the compositing factor), and the NSW demographic profile from the 2011 census [30].

**Table 4:** Sample comparisons to the latest population profile for NSW

**Demographic group**		**Landline frame**		**Mobile frame**		**Landline plus mobile only**		**Both frames combined #**		**2011 Census**
		**Total (%)**	**p-value**	**Total (%)**	**p-value**	**%**	**p-value**	**%**	**p-value**	
Age groups	0-15	14.1	**<0.001**	11.4	**0.03**	13.4	**<0.001**	12.1	**0.01**	20.5
	16-24	4.1		12.4		5.8		7.3		11.6
	25-34	5.6		23.1		10.2		13.2		13.6
	35-44	7.6		15.0		8.2		9.9		14.1
	45-54	13.0		16.9		12.7		13.4		13.8
	55-64	21.6		12.9		19.8		17.4		11.7
	65-74	18.4		6.5		16.4		14.1		7.8
	75-high	15.6		1.7		13.5		12.5		6.9
Sex	Male	38.9	**0.04**	48.4	0.85	40.1	0.07	42.8	0.20	49.3
	Female	61.1		51.6		59.9		57.2		50.7
Aboriginality	Aboriginal	2.2	0.86	2.6	0.94	2.6	0.96	2.6	0.96	2.5
	Non-Aboriginal	97.8		97.4		97.4		97.4		97.5
Country of birth	Australia	79.4	**0.02**	64.9	0.42	77.1	0.07	73.4	0.30	68.6
	Overseas	20.6		35.1		22.9		26.6		31.4
Marital status	Married	54.1	**<0.001**	54.0	0.76	51.3	**0.01**	51.5	0.08	49.4
	Widowed	13.7		3.1		12.2		11.1		5.8
	Separated	4.0		3.3		3.9		3.7		3.1
	Divorced	12.3		7.1		11.7		10.2		8.3
	Never married	15.9		32.5		20.9		23.5		33.4
Income*	< $20,000	24.0	**0.02**	12.0	**0.04**	23.4	**0.02**	21.9	0.05	13.7
	$20,001-$40,000	19.8		15.4		19.2		18.5		19.8
	$40,001-$60,000	15.1		14.9		15.3		14.7		16.9
	$60,001-$80,000	10.4		14.0		10.8		11.2		19.8
	$80,000 plus	30.8		43.7		31.3		33.7		29.8

*Notes*: ^#^ Calculation numbers for combined frame = ((S_a_+λS_ab_^A^)+ (S_b_+(1-λ)S_ab_^B^) where S =sample; λ=overlap adjustment (set to 0.5); _A_ landline sample frame; _B_ denotes mobile sample frame; _a_ landline-only phone users; _b_ mobile-only phone users; _ab_ denotes both mobile phone and landline users.

* Census income information was converted from weekly income to annual income for the comparison.

χ2 testing, setting the significance level of p<0.05, was used for the comparisons between the sample demographic categories and the population profile (2011 census).

As shown in Table 4 the NSW demographic profile was significantly different to respondents: from the landline frame for age group (p<0.001), sex (p=0.037), country of birth (p=0.02), marital status (p<0.001) and income (p=0.015); from the mobile frame for age group (p=0.03) and income (p=0.04); from the landline frame plus mobile-only phone respondents for age group (p<0.001), marital status (p=0.01) and income (p=0.02); and from the combined frame for age group (p=0.01).

## Discussion

When mobile phone numbers were included in the first quarter of 2012 into the NSWPHS using an overlapping dual-frame design, 3395 interviews were completed with just under two thirds from the landline frame and just over one from the mobile frame. Interviews that resulted from the mobile frame, compared to the landline frame, were slightly shorter, cost 2.3 times more for each completed interview and required more telephone numbers to obtain a contact, eligible contact and an interview. Response, contact and co-operation rates were similar between frames. Overall combined response, contact and cooperation rates were 33.1%, 65.1% and 72.2% respectively. As expected from previous research [19-23], the demographic profile of the mobile-only phone respondents differed most (more that were young, males, Aboriginal and Torres Strait Islanders, overseas born and single) compared to the landline frame responders. The demographic profile of respondents from the two frames combined, with appropriate overlap adjusted, was most similar to the latest NSW population profile.

The inclusion of the mobile phone number was logistically very challenging with the biggest challenge being the lack of geography on the mobile frame which resulted in more time and resources being spent on calling ineligible numbers (persons who reside outside NSW). The inclusion of mobile phone numbers in the NSWPHS however is still cost-effective because of the additional interviews that were conducted with young people, Aboriginal and Torres Strait Islanders and people who were born overseas resulting in a more representative sample. This however may not be the case for smaller states where the cost of excluding ineligible (out of state) persons may be prohibitive.

As this study is mainly descriptive there is a need to further examine if using a different overlap adjustment factors would have impacted on the results. Further work also needs to occur with the sample frame provider to minimise the number of invalid and ineligible number (predominantly business numbers) to improve the efficiency of the data collection.

Early results are now becoming available from stand-alone surveys of the Australian population that are including mobile phone numbers using various designs [31-33] and so we are slowly getting more experience in Australia on conducting RDD surveys with mobile phone augmentation. There is still a need for more detailed methodologies to be provided. So hopefully this study, and the work we are undertaking on weighting strategies for the NSWPHS and an examination of the impact of the design change on the time series, will contribute to a better understanding of how to conduct RDD surveys with mobile phone augmentation in Australia.

## Conclusions

The inclusion of the mobile phone numbers, through an overlapping dual-frame design, did not impact negatively on response rates or data collection, and although costing more the design was still cost-effective because of the additional interviews that were conducted with young people, Aboriginal and Torres Strait Islanders and people who were born overseas resulting in a more representative overall sample.

## Abbreviations

AAPOR: American Association For Public Opinion Researchers; ACMA: Australian Communications And Media Authority; CATI: Computer assisted telephone interviewing; RDD: Random digit dialling; NSWPHS: New South Wales Population Health Survey.

## Competing interests

The authors declare that they have no competing interests.

## Authors' contributions

MLB developed the overall concepts and planned the study; undertook the analysis and co-wrote the methods and results, wrote the introduction and discussion and finalised the manuscript. JJVR developed and managed the data collection, co-wrote the methods and results, and commented on drafts of the manuscript. DGS provided development and analysis advice and commented on drafts of the manuscript. SVT provided overall support for the study and commented on drafts of the manuscript. All authors read and approved the final manuscript.

## Authors' information

MLB is a PhD student with the Centre for Statistical and Survey Methodology, University of Wollongong, Wollongong, Australia.

## Pre-publication history

The pre-publication history for this paper can be accessed here:

http://www.biomedcentral.com/1471-2288/12/177/prepub
